# Supplementary material for: Health inequity on access to services in the ethnic minority regions of Northeastern Myanmar: a cross-sectional study
Source: BMJ Open. 2017 Dec 14;7(12):e017770. doi: 10.1136/bmjopen-2017-017770 (PMC5735411; doi:10.1136/bmjopen-2017-017770)
Supplement: Author's manuscript [file bmjopen-2017-017770.draft_revisions.pdf]

BMJ Open is committed to open peer review. As part of this commitment we make the peer review history of every article we publish publicly available.

When an article is published we post the peer reviewers' comments and the authors' responses online. We also post the versions of the paper that were used during peer review. These are the versions that the peer review comments apply to.

The versions of the paper that follow are the versions that were submitted during the peer review process. They are not the versions of record or the final published versions. They should not be cited or distributed as the published version of this manuscript.

BMJ Open is an open access journal and the full, final, typeset and author-corrected version of record of the manuscript is available on our site with no access controls, subscription charges or pay-per-view fees (http://bmjopen.bmj.com).

If you have any questions on BMJ Open's open peer review process please email editorial.bmjopen@bmj.com

BMJ Open

Health inequity in the ethnic minority regions of Northeastern Myanmar

Journal:	*BMJ Open*
Manuscript ID	bmjopen-2017-017770
Article Type:	Research
Date Submitted by the Author:	18-May-2017
Complete List of Authors:	Tang, Kun; Peking University, Department of Global Health<br>Zhao, Yingxi; Peking University, ;<br>Li, Bolun; Peking University<br>Zhang, Siqiao
**Primary Subject Heading**:	Global health
Secondary Subject Heading:	Global health, Health services research, Public health, Health policy
Keywords:	myanmar, conflict, ethnic minority, health equity

SCHOLARONE™
Manuscripts

Health inequity in the ethnic minority regions of Northeastern Myanmar

Kun Tang[1*#], Yingxi Zhao[2#], Bolun Li[2], Siqiao Zhang[3]

1 Department of Global Health, Peking University School of Public Health, Beijing, China
2 Institute for Medical Humanities, Peking University Health Science Center, Beijing, China
3 Peking University School of Public Health, Beijing, China
* Correspondence: tangkun@hsc.pku.edu.cn; Tel.: +86-10-82805951;
# These authors contribute equally to the study

Kun Tang: Department of Global Health, Peking University School of Public Health, Beijing, China tangkun@hsc.pku.edu.cn
Yingxi Zhao: Institute for Medical Humanities, Peking University Health Science Center, Beijing, China zhaoyingxi@pku.edu.cn
Bolun Li: Institute for Medical Humanities, Peking University Health Science Center, Beijing, China 450925110@qq.com
Siqiao Zhang: Peking University School of Public Health, Beijing, China 896481989@qq.com

For peer review only

Abstract

Objectives: To analyze health inequity in the ethnic regions of Northeastern Myanmar from three aspects: geographic barrier, gender-based disparity and financial burden of health services.

Setting: A multistage-stratified random cluster survey was conducted in Shan State Special Region 2 and Eastern Shan State Special Region 4 of Northeastern Myanmar in 2016, and a total number of 774 households were included.

Participants: A total number of 4,235 participants were recruited in the survey

Primary and secondary outcome measures: geographic distance, gender and household income and inpatient/outpatient service utilization

Results: Residents living within 5 km of any healthcare facilities paid more outpatient visits (90.06 visits per thousand population) in the past two weeks, compared to those living between 5-20 km and over 20 km (54.84 and 54.02 per thousand population). Similar but less significant difference was found for inpatient service use. Adult with a household income of above 720 USD/year were more likely to seek for outpatient service (OR=1.43, 95%CI 0.98-2.10), compared to those with an annual income of <360 USD. After adjusting for other covariates, female adults were less likely to seek inpatient treatment (OR=0.55, 95%CI 0.35-0.84) and outpatient services (OR=0.86, 95%CI 0.64-1.15), compared to males.

Conclusions: Geographic barrier, gender-based disparity and financial burdens significantly restrict the ethnic people's access to healthcare facilities. Tackling health inequity in the Myanmar border areas requires a better primary healthcare system, proper financial protection mechanisms and special focus on women.

Keywords: Myanmar, conflict, health equity, ethnic minority

Strengths and limitations of this study

- The present study is the largest and one of the few studies of health inequity targeted in Myanmar-China border area, where health data is severely scarce.
- We found that health inequity was a major issue leading to a poor health status in the study areas, and such an inequity in health was attributable to poverty, a lack of financial protection, geographic barriers and gender-based disparity.
- The cross-sectional nature of this study does not allow for causal inference to provide more robust evidence

For peer review only

1. Introduction

Since Myanmar's signing of Panglong agreement in 1947 and its independence in 1948, ethnic minority regions have been torn by conflicts between the central government and ethnic armed organizations (EAOs), the latter of which seek for a full local autonomy under federalism. Continuous military conflicts in Kachin, northern Shan as well other states has led to some 120,000 internally displaced civilians in the past years and more than 200 villages have been destroyed [1]. Despite the 21st century peace talk led by Aung San Suu Kyi in September 2016, which aimed to stop conflict and achieve peace that has eluded the country for three-quarters of a century, recent air strikes and fights across Shan State's Muse and Kutkai townships in November 2016 again resulted in dozens of casualties, 2,600 people displaced and over 3,000 people fled across the border into China [2]. The ongoing conflict led to notoriously weak health statistic: a 2014 malaria survey along the Myanmar-China border suggested an incidence of 8.70 per thousand [3], almost triple Myanmar's national average [4] - not to mention that Myanmar's health situation is already in the lower range among Southeast Asian countries in the Greater Mekong Sub-region.

Health equity has always been the key focus of public health at all levels, while issues around health equity in conflict-affected fragile states have been especially difficult to tackle due to constraints in data and a lack of credible evidence. Beside a cross-country health inequity armed conflicts could also be a primary driver of health inequities within a country, as evidenced by reduced coverage of preventive intervention and high disease mortality in violence-reported areas [5]. The infant mortality and under-five mortality in Eastern Myanmar conflict regions were 94.20 and 141.90 per thousand live births in 2013, around double to triple the statistics of central Myanmar, and similar to those of Somalia [6]. Various socio-economic determinants potentially contributed to the health inequity in Myanmar's conflict areas, such as geographic disparities in access to healthcare, gender-based inequity, inequities due to displacement status, financial barrier, as well as reduced health management and service capacity [7].

Despite of a deteriorating health status in the Myanmar-China border area, the issue of health inequity in Northeastern Myanmar has largely been missing in literature. The existing literature mostly focused on the Thai-Myanmar border, where the social and political context, ethnicity composition, economic condition and health status are largely different. So far, little has been reported about these particular conflict regions of Kachin State and Eastern Shan State. This study aims to analyze health inequity in the ethnic minority regions of Northeastern Myanmar, with data from a population-representative cross-sectional survey conducted in 2016. Through describing the health service utilization in the study regions and analyzing its association with various socio-demographic indicators, this study reports low health inequity from three aspects: geographic barrier, gender-based disparity and financial burden of health services.

For peer review only

2. Methods

Study Design

A survey using multistage-stratified random cluster sampling scheme was conducted in Shan State Special Region 2 (Wa region) and Eastern Shan State Special Region 4 (SR4) of Northeaster Myanmar in March, 2016. To ensure the population representativeness, randomization procedures were applied through multistage: at the first stage of sampling, 75 village-level units were randomly chosen with the probability proportional to scale (PPS) from a sampling frame containing all village-level units under seven townships in Wa region and SR4. At the second stage, all households in a village were listed, from which ten households were randomly chosen within each village using geographically randomization approach – after one household was picked, every other household was chosen in a geographically clockwise sequence. A total number of 774 households and 4,235 participants were eventually recruited in the survey. The household data included detailed information of the respondent family and every individual response within the family. The survey questionnaire contained detailed information on basic demographics, household, health status, health care, employment, household economy (income, consumption and wealth), health service utilization and health knowledge. All data were collected by face-to-face computer-aided personal interviews (CAPI) and input into Epidata. The questionnaire was repeatedly tested to ensure high data quality. Ethical approval was obtained from Zhejiang University. Written informed consent was obtained from all participants.

Exposure and Outcome Variables

In this study, the main outcome variables included outpatient and inpatient information. Outpatient service utilization included the frequency of outpatient visit during the past year (none, 1, or ≥2), place of outpatient visit (i.e. private clinics, rural health centers, township hospitals, healthcare facilities in China, or others), and average cost for outpatient visits; whereas inpatient service utilization included the frequency of inpatient visit during the past two weeks (none, 1, or ≥2), reasons for inpatient visit (disease, poisoning and injury, delivery, or others), place of inpatient visit (private clinics, rural health centers, township hospitals, healthcare facilities in China, or others), and average cost for inpatient visits.

Exposure variables included age, gender, education level (i.e. illiterate, 0-5 years of education, ≥6 years, and under 14 years old - individuals aged below 14 were categorized as “under 14 years old” due to ongoing education), ethnicity (i.e. Dai, Anni, Wa, Lahu, Kokang, Jingpo, Burman, Blang, etc.), occupation (i.e. government/military, engineering, business and agriculture, others, and under 14 years old - individuals aged below 14 were categorized as “under 14 years old” due to ongoing education), physical distance from home to health care facilities (i.e. <5km, 5 to 20 km and >20 km), household income (i.e. 0 to 360 US Dollars, 360 to 720 US Dollars, and > 720 US Dollars) and family general nutrition status (i.e. enough food supply or lacking in food supply).

For peer review only

Statistical analysis

Selected demographic and socioeconomic characteristics were described separately for male and female. The distribution of socio-demographic, unadjusted means with standard deviations (SD) for continuous variables and unadjusted proportions for categorical variables were presented. Health service utilization including the frequency, reasons and places for inpatient and outpatient visit were separately presented by region and gender. The average cost for inpatient/outpatient visit was presented by different types of healthcare facilities.

To analyze the association between physical distance to nearest healthcare facility and inpatient and outpatient behavior, linear regression was performed and adjusted means of frequency to inpatient and outpatient service per thousand population by physical distance were presented. All linear models were commonly adjusted for covariates, including age, gender, household income and ethnicity.

The association between each exposure variable and inpatient and outpatient utilizations was analyzed using logistics regression. Odds ratios (OR) of using inpatient/outpatient services were reported within each gender, income level, education level, and physical distance to nearest facility and study region. As the population under the age of 14 did not report education level, results were separately reported for population below and above 14 years of age. All statistical analyses were performed using SAS version 9.3 (SAS Institute Inc., Cary, North Carolina, USA), and test results were reported significant at 0.05 level.

3. Results

Baseline participants' characteristics stratified by regions are presented in Table 1. Overall, of the 4,235 participants included in the analyses, 3,246 (76.60%) were from Wa region, and respectively 1,638 (50.46%) in Wa region and 500 (50.56%) in SR4 were male. At the time of the survey, 41.74% of the Wa region's population was under the age of 14, and 32.56% of the Wa population was between 15 and 34 years of age. In comparison, 31.75% and 34.88% of SR4 population was under 14 years and between 15 and 34 years, respectively. 44.31% and 45.91% of Wa and SR4 resident were illiterate. 29.70% of the Wa population and 73.00% of the SR4 population reported an annual income of above 720 USD, while majority of the population in Wa region (44.45%) reported an annual household income of less than 360 USD. In Wa region, most of the population (82.01%) was Wa ethnicity, while SR4 constituted a more complex variety of ethnicities. Half of the households in SR4 reported a physical distance to the nearest healthcare facilities within 5 km, while about 34.54% of the surveyed household in Wa region reported a physical distance to the nearest healthcare facilities over 20 km.

For peer review only

	Wa Region (N= 3,246)	Eastern Shan State SR4 (N= 989)
**Age, years**		
0-14	1355(41.74)	314(31.75)
15-34	1057(32.56)	345(34.88)
⩾35	834(25.70)	330(33.37)
**Sex**		
Male	1638(50.46)	500(50.56)
**Education**		
Under 14 years old	1355(41.74)	314(31.75)
Illiterate	1428(44.31)	454(45.91)
0-5 years	313(9.64)	147(14.86)
≥6 years	140(4.31)	74(7.48)
**Household Income**		
<360 USD	1443(44.45)	82(8.29)
360-720 USD	839(25.85)	185(18.71)
>720 USD	964(29.70)	722(73.00)
**Ethnicity**		
Wa	2662(82.01)	—
Dai	141(4.34)	352(35.59)
Aini	—	158(15.98)
Others	443(13.65)	479(48.43)
**Occupation**		
Under 14 years old	1355(41.74)	314(31.75)
Government/military	105(3.23)	37(3.74)
Engineering, business and agriculture	1670(51.45)	598(60.47)
Others	115(3.57)	40(4.04)
**Types of drinking water**		
Well water	17(0.52)	140(14.16)
Hose water	806(24.83)	685(69.26)
Others	2423(74.65)	164(16.58)
**Physical Distance to Nearest Healthcare Facility**		
<5 km	1080(33.27)	487(49.24)
5-20 km	1045(32.19)	355(35.90)
>20 km	1121(34.54)	147(14.86)
**Nutrition status**		
Lacking in food supply	1187(36.57)	171(17.29)

Table 1. Basic Characteristics of 4,235 Participants by Region

Source: author analysis

Values are N (%) unless otherwise specified.

Table 2 showed the characteristics of healthcare service utilization in the study regions. Only 2.16% of Wa residents and 4.65% of SR4 residents reported having received inpatient treatment in the past year. Similarly, only 6.56% of Wa residents and 6.88% of SR4 residents reported having paid outpatient visit in the past two weeks. Having disease conditions was the major reason for both inpatient and outpatient service utilizations in Wa region and SR4. While more residents in Wa region preferred private clinic for outpatient services (61.03%)

For peer review only

and township hospitals for inpatient services (45.71%), more SR4 residents chose healthcare facilities in China for inpatient service (39.13%). Of those who used health services, males were more likely to choose private clinics or health services in China. As is shown in Figure 1, average cost for outpatient and inpatient visit in China's health facilities was 449.09 USD and 1414.58 USD, respectively, which was two to three times higher than that in township hospitals (205.28 and 411.37 USD). The average costs for primary health care provided by private clinics and rural health centers were much lower, but the utility of these services were also relatively low.

	**Wa Region (N=3,246)**	**SR4 (N=989)**	***p***	**Male (N=2,138)**	**Female (N=2,097)**	***p***
**Inpatient in the past year**			<0.001			0.0180
Yes	70 (2.16)	46 (4.65)		46 (2.15)	70 (3.23)	
No	3,176 (97.84)	943 (95.35)		1092 (97.85)	2027 (96.77)	
**Reason for inpatient among all inpatient population**			<0.001			0.0402
Disease	64 (91.43)	39 (84.78)		45 (97.83)	58 (82.86)	
Poisoning and injury	2 (2.86)	1 (2.17)		1 (2.17)	2 (2.86)	
Delivery	4 (5.71)	6 (13.05)		-	10 (14.28)	
**Place for inpatient among all inpatient population**			0.0003			0.8946
Rural health centers	1 (1.43)	3 (6.52)		1 (2.17)	3 (4.29)	
Township hospitals	32 (45.71)	8 (17.40)		14 (30.43)	26 (37.14)	
Private clinics	19 (27.14)	6 (13.04)		11 (23.91)	14 (20.00)	
Healthcare facilities in China	13 (18.57)	18 (39.13)		13 (28.26)	18 (25.71)	
Others	5 (7.15)	11 (23.91)		7 (15.23)	9 (12.86)	
**Outpatient in the past two weeks**			0.7286			0.2234
Yes	213 (6.56)	68 (6.88)		132 (6.17)	149 (7.11)	
No	3,033 (93.44)	921 (93.12)		2006 (93.83)	1948 (92.89)	
**Place for outpatient among all outpatient population**			0.0003			0.2113
Rural health centers	15 (7.04)	6 (8.82)		10 (7.57)	11 (7.38)	
Township hospitals	47 (22.07)	23 (33.82)		30 (22.73)	40 (26.85)	
Private clinics	130 (61.03)	23 (33.82)		77 (58.33)	76 (51.01)	
Healthcare facilities in China	9 (4.23)	14 (20.60)		11 (8.33)	12 (8.05)	
Others	12 (5.63)	2 (2.94)		4 (3.04)	10 (6.71)	

Table 2. Characteristics of health service utilization by region and sex

Source: author analysis

Values are N (%) unless otherwise specified. P-value refers to comparison between Wa region and SR4, or between males and females

Figure 2 (a) and (b) described the adjusted mean frequency of inpatient/outpatient visits per thousand population by physical distance. In general, residents living within 5 km paid more

For peer review only

outpatient visits to healthcare facilities in the past two weeks, compared with residents living between 5-20 km and over 20 km (90.06, 54.84 and 54.02 per thousand population per two weeks). In comparison, residents living within 5 km and between 5-20 km used slightly more inpatient services than did residents living above 20 km in the past year. Figure 2 (c) and (d) illustrate the gender difference in outpatient visit. The average frequency of outpatient service utility decreased from 93.15 visits per thousand population in those living within 5 km to the nearest hospital/clinic, to 50.01 in those living over 20 km in males; and in females, the average frequency decreased from 83.63 in those living within 5 km to 56.96 in those living over 20 km. Shown in Figure 2 (e) and (f), SR4 population reported a larger number of outpatient visits within each distance level than those in Wa region, the mean frequency of outpatient visit for those living within 5 km was 89.85 per thousand population in the past two weeks in Wa region and 173.09 in SR4. For those living above 20 km, the mean frequency were 59.77 and 91.57 per thousand population for Wa region and SR4, respectively.

	**Outpatient**		**Inpatient**	
	**Age**<14<br>OR (95% CI)	**Age≥14**<br>OR (95% CI)	**Age**<14<br>OR (95% CI)	**Age≥14**<br>OR (95% CI)
**Sex**				
Male	1.00	1.00	1.00	1.00
Female	1.06(0.66-1.72)	0.86 (0.64-1.15)	1.43 (0.55-3.73)	0.55 (0.35-0.84)
**Household Income**				
<360 USD	1.00	1.00	1.00	1.00
360-720 USD	3.43(1.73-6.83)	1.04 (0.69-1.55)	1.71 (0.51-5.76)	1.53 (0.82-2.86)
>720 USD	2.95 (1.48-5.86)	1.43 (0.98-2.10)	1.04 (0.28-3.85)	1.99 (1.09-3.64)
**Distance to nearest healthcare facilities**				
<5 km	1.00	1.00	1.00	1.00
5-20 km	0.37 (0.21-0.66)	0.67 (0.47-0.95)	0.77 (0.26-2.28)	1.07 (0.66-1.73)
>20 km	0.17 (0.07-0.38)	0.79 (0.55-1.13)	0.45 (0.12-1.74)	0.75 (0.43-1.32)
**Education**				
Illiterate	/	1.00	/	1.00
0-5 years	/	0.55 (0.35-0.86)	/	1.12 (0.66-1.90)
≥6 years	/	0.64 (0.36-1.15)	/	0.76 (0.34-1.74)
**Region**				
Wa	1.00	1.00	1.00	1.00
SR4	0.42 (0.17-1.08)	1.04 (0.64-1.68)	3.35 (0.45-25.00)	1.43 (0.75-2.71)

Table 3. Odds Ratio of the association between selected variables and health seeking behavior in Wa region and Eastern Shan State SR4*

Source: author analysis

*All models commonly adjusted for sex, household income, physical distance to nearest healthcare

For peer review only

facility, education level and ethnicity.

Table 3 showed the association between selected socio-demographic indicators and inpatient/outpatient behavior. After adjusting for other covariates, females above 14 were less likely to seek inpatient treatment compared to males of the same age group (OR=0.55, 95%CI 0.35-0.84), and slightly unlikely to seek outpatient treatment (OR=0.86, 95%CI 0.64-1.15), while no such association was found among children of both sexes aged below 14 years. Longer distance to the nearest healthcare facilities was associated with a decreased likelihood of inpatient/outpatient utilizations in all age groups. Household income was positively associated with outpatient behavior. Those aged above 14 years with an annual household income of above 720 USD were more likely to seek outpatient service (OR=1.43, 95%CI 0.98-2.10) compared to those with an income of less than 360 USD per year. A similar association was also observed in inpatient visits between the two income groups (OR=1.99, 95%CI 1.09-3.64). As for those aged below 14, a household income between 360-720 USD and above 720 USD both increased the odds of seeking outpatient services (OR=3.43, 95%CI 1.73-6.83, and OR=2.95, 95%CI 1.48-5.86, respectively). As for education level, longer year of education was associated with a reduced odds of outpatient visits (OR=0.64 and 0.55 for those with an education of longer than 6 years and between 0-5 years).

4. Discussion

The present study is the largest and one of the few studies of health inequity targeted in Myanmar-China border area. In the analysis of a population-representative survey in two regions, we found that health inequity was a major issue leading to a poor health status in the study areas. Such an inequity in health was attributable to poverty, a lack of financial protection, geographic barriers and gender-based disparity.

There are several potential limitations of the present study: firstly, this study's cross-sectional nature does not allow for causal inference to provide more robust evidence; secondly, the interviews were conducted by an international NGO that provided public health service to the regions, potential interviewer bias and respondent's recall bias need to be taken into account. Nonetheless, the present findings from the population-representative survey provides a good insight of service utilization in Northeastern Myanmar border area, where health data is severely scarce.

a) Inequitable health due to poverty and a lack of financial protection

Our study found that socio-economic condition, represented by household income is strongly associated with inpatient and outpatient service utilization in this region. Besides the fact that generally low economic status leads to the extreme low service utilization in both inpatient and outpatient services, the financial burden of health services also diverges patients to different levels of healthcare facilities. People with higher household income tend to visit

For peer review only

healthcare facilities more frequently or choose to use healthcare facilities in China, the user fee of which is generally much higher than those in the ethnic regions. In contrast, most of the population with lower household income tend to use private clinics, and in most occasions, small pharmacies, where services are more likely to be provided by uncertified and unqualified health practitioners or pharmacists.

Out-of-pocket expenditure is still a major source of total health expenditure in Northeastern Myanmar border areas, and the proportion is estimated to be higher than that of central Myanmar, which was above 90% in 2014 [8]. Considering that most of the households' annual income is less than 1 dollar a day in Wa region, an outpatient visit to township hospital would cost over half of their annual household income. Besides, the local health system's bias towards civilians exacerbates inequity of health service. Both Wa region and SR4 cover the outpatient/inpatient fee for military personnel, and reimburse 50% of the fee for soldiers' family members. However, no financial coverage is provided for civilian and children. On the other hand, in the hospitals and rural health centers opened by central Myanmar government, many health professionals charge informal payments or diverge the patients to private hospital/clinics. This simply poses a financial barrier to access government-funded healthcare services among the poor [7]. In both Myanmar government health facilities and ethnic health organizations, high fee-for-service may bring about catastrophic health care expenditures, and made healthcare services unaffordable to patients with lower socio-economic status. Tackling the financial barriers is perhaps one of the utmost important issues in improving health service utilization in Northeastern Myanmar.

b) Geographic disparities

Our study found that physical distance to the nearest healthcare facilities could largely influence people's frequency of inpatient and outpatient use in the study regions. The result is consistent with most empirical studies as the patients might have difficulty in accessing the hospitals and clinics if the physical distance is far [9, 10]. In a region with uncertainty of security, degradation of transportation infrastructure and even unstable external environment including extreme weather, health equity is much deteriorated due to geographic barriers. Nonetheless, our study suggests that geographic distance mainly influences outpatient visit, and access to primary health care in particular. There is no evidence that physical distance influences inpatient service utilization in these regions, probably because the inpatient visits are often closely correlated with severe disease conditions in which people must be transferred to health facilities for medical attention.

Whilst the lack of transportation – people usually have to walk to the clinics especially in rainy seasons – obstructs the accessibility to healthcare facilities, the uneven geographical distribution of healthcare facilities is another major contributing factor to the inequitable situation. Over 50% of the population does not have access to healthcare facilities within 5 km in both regions. There are only eight general hospitals, 60 rural health centers and less than 100 private clinics (most of them are private pharmacies) in the region serving the 0.6

For peer review only

million population. Most of these healthcare facilities locate in capital regions, leaving the suburban and rural areas a vacuum of healthcare services. In the event of conflict, most of the Burmese doctors and private doctors from China would choose to suspend their services and retreat to safer regions. The unbalanced service distribution, exacerbated by conflict, further aggravate health equity in the ethnic conflict regions in Northeastern Myanmar.

c) Gender-based inequities

In the study population of those above 14 years old, females were less likely to seek inpatient treatment compared with males. Women are usually recognized as a particularly vulnerable group as they constitute majority of internally displaced population and refugees in conflict settings [7]. Rural and ethnic minority women in Myanmar, in particular, could barely achieve equal rights with men. A series of cultural, economic, legal and political factors may perpetuate gender-based inequity to healthcare services in these regions, including but not limited to cultural norms, the low social status of female, and female's financial dependence on male [11]. Despite international NGO's long-term efforts on maternal and child health, very few women enjoyed hospital-facilitated delivery, accounting for only 14.28% of all inpatient service for female, which was much lower than Myanmar's national average 36.2% [12]. Empowering women in ethnic regions is an important approach not only to improve general health status, but also to overcome social and cultural barriers to gender disparity.

This is one of the first studies to explore the different aspects of health inequity, including geographic barrier, gender-based disparity, as well as financial burden of health services, in border areas of northeastern Myanmar. The findings suggest that all these factors strongly restrict local people's access to healthcare services. Tackling health inequity in the Myanmar border areas requires a better primary healthcare system to fill the gap of geographic disparity, a better financial protection mechanism to build in the current health systems, as well as a special focus on women and girls. However, prolonged armed conflicts not only complicate health service delivery through the already weak health infrastructures, but also deteriorate the gender inequity.

Empirical evidence suggests that countries/regions emerging from war and conflict usually end up having weak or even non-operational health systems, which further expose them to emerging and re-emerging epidemics [13]. The sole focus on vertical programs from international development assistance could serve for short-term needs but may also further delay the strengthening of local health system. While the national ceasefire agreement (NCA) has encountered huge challenge [14], the nationwide peace process and the new "21st Century Panglong Conference" suggests new opportunities for health system strengthening in the ethnic regions in northeastern Myanmar. With additional support and financial resources from national government and international agencies, a comprehensive package of primary health care system should be established to reduce health inequity for ethnic peoples.

For peer review only

Reference

1. Weng, Lawi (2014) 200 Kachin Civilians Flee Fighting Near Hpakant. The Irrawaddy. Available: http://www.burmanet.org/news/2014/08/14/the-irrawaddy-200-kachin-civilians-flee-fighting-near-hpakant. Accessed Dec 22, 2016.
2. Myanmar Times (2016) Air Strikes Reported as Shan State Conflict Spreads. Available: http://www.mmtimes.com/index.php/national-news/23870-air-strikes-reported-as-shan-state-conflict-spreads.html. Accessed Dec 22, 2016.
3. Wang R, Dong J, Xia Z, Cai T, Zhang Q & Zhang Y, et al. (2016) Lessons on malaria control in the ethnic minority regions in northern myanmar along the china border, 2007–2014. Infectious Diseases of Poverty, 5(1), 95.
4. Mu T, Sein A, Kyi T, Min M, Aung N, & Anstey N, et al. (2016) Malaria incidence in myanmar 2005–2014: steady but fragile progress towards elimination. Malaria Journal, 15(1), 503.
5. Victora C, Fenn B, Bryce J, & Kirkwood B. (2005) Co-coverage of preventive interventions and implications for child-survival strategies: evidence from national surveys. Lancet, 366(9495), 1460.
6. Asia foundation (2015) The Long Road to recovery Ethnic and Community-Based Health Organizations Leading to Better Health in Eastern Burma. Available: http://reliefweb.int/sites/reliefweb.int/files/resources/The%20Long%20Road%20to%20Recovery%202015_Eng.pdf. Accessed Dec 22, 2016.
7. Bornemisza O, Ranson M, Poletti T, & Sondorp E. (2010) Promoting health equity in conflict-affected fragile states. Social Science & Medicine, 70(1), 80-88.
8. World Health Organization (2014) The Republic of the Union of Myanmar Health System Review. Available: http://www.wpro.who.int/asia_pacific_observatory/hits/series/myanmar_health_systems_review.pdf. Accessed Dec 22, 2016.
9. Awoyemi T, Obayelu O, & Opaluwa H. (2010) Effect of distance on utilization of health care services in rural kogi state, Nigeria. J Hum Ecol, 35(1): 1-9.
10. Buor D. (2003). Analysing the primacy of distance in the utilization of health services in the ahafo-ano south district, Ghana. The International Journal of Health Planning and Management, 18(4), 293–311.
11. United Nations High Commissioner for Refugees (1999) Reproductive health in refugee situations: An inter-agency field manual. Available: http://www.who.int/reproductivehealth/publications/emergencies/field_manual_rh_humanitarian_settings.pdf. Accessed Dec 22, 2016.
12. Myanmar Ministry of Health (2010) Myanmar Multiple Indicator Cluster Survey 2009 – 2010. Available: https://www.unicef.org/myanmar/MICS_Myanmar_Report_2009-10.pdf. Accessed Mar 3, 2017.
13. Bisika T (2010) Health systems strengthening in conflict situations. East Afr J Public Health, 7(3), 277-281.

For peer review only

14. Asian Tribune (2015) Myanmar's NCA seems half-done missing some signatories Available: http://www.asiantribune.com/node/88058. Accessed Mar 3, 2017.

Contributorship statement

TK, ZYX and LBL made the statistical analysis and drafted the manuscript; ZSQ revised the article for important intellectual content and wrote part of the article. All authors read and approved the final manuscript.

Data availability

The dataset supporting the conclusion of this article is available from a third party. Please contact the corresponding author to apply to gain access to the relevant data.

Data sharing statement

No additional data available

Funding

The authors declare no funding source.

Competing interests

The authors report no conflict of interest.

Figure 1. Average cost for inpatient and outpatient visit in USD.

Source: author analysis

Figure 2. Adjust means of frequency to inpatient and outpatient service by physical distance*

Source: author analysis

*Adjusted for age, gender, household income and ethnicity

For peer review only

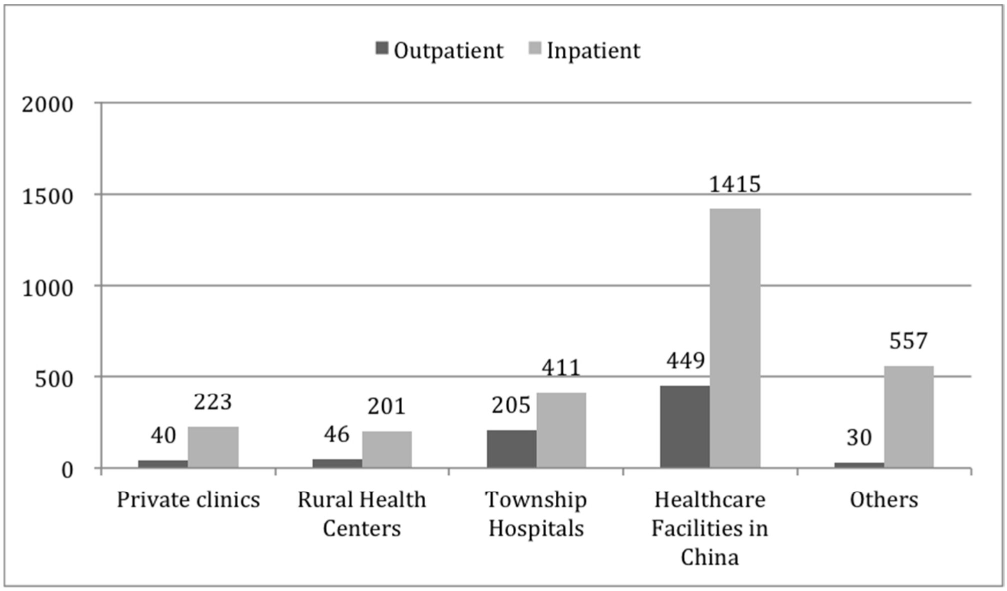

146x85mm (150 x 150 DPI)

review only

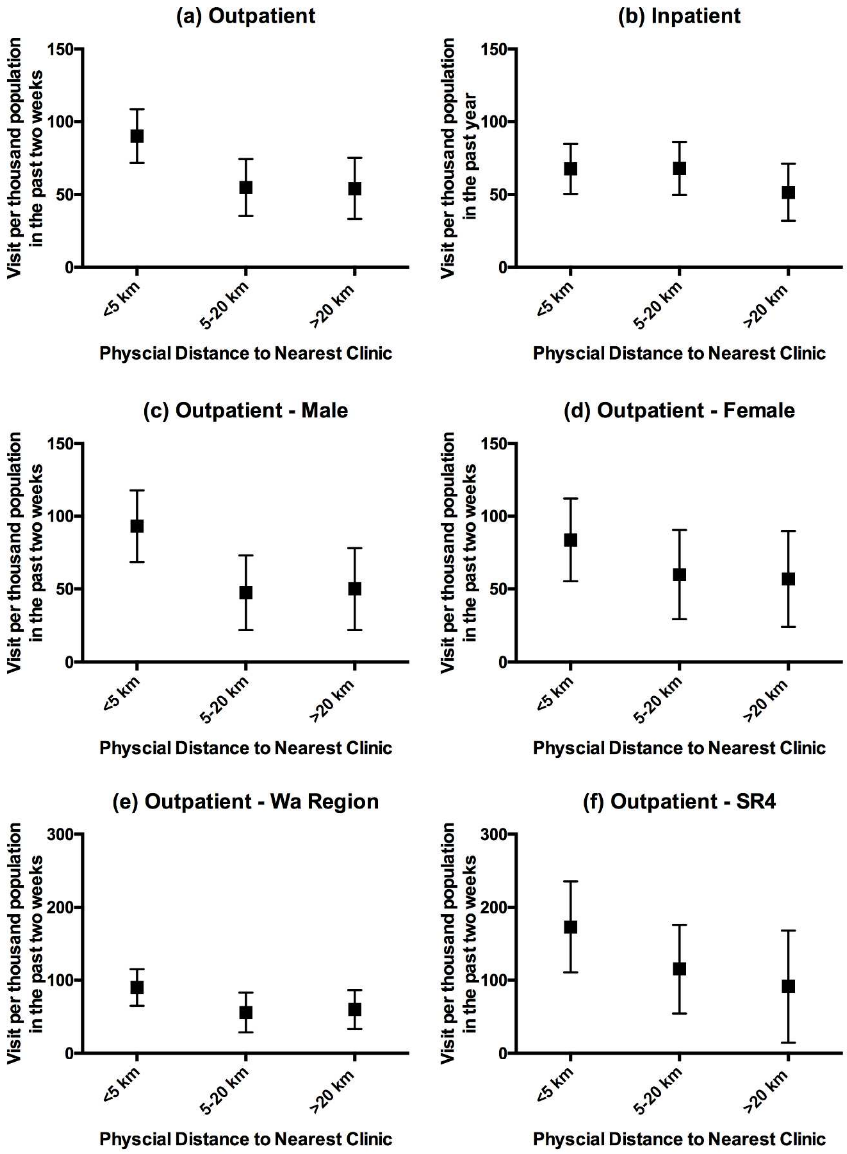

200x280mm (300 x 300 DPI)

BMJ Open

Health inequity on access to services in the ethnic minority regions of Northeastern Myanmar: A cross-sectional study

Journal:	*BMJ Open*
Manuscript ID	bmjopen-2017-017770.R1
Article Type:	Research
Date Submitted by the Author:	04-Oct-2017
Complete List of Authors:	Tang, Kun; Peking University, Department of Global Health<br>Zhao, Yingxi; Peking University, Institute for Medical Humanities; University of Washington, Department of Global Health<br>Li, Bolun; Peking University, Institute for Medical Humanities<br>Zhang, Siqiao; Peking University, School of Public Health<br>Lee, Sung Hoon; Peking University School of International Studies
**Primary Subject Heading**:	Global health
Secondary Subject Heading:	Global health, Health services research, Public health, Health policy
Keywords:	myanmar, conflict, ethnic minority, health equity

SCHOLARONE™
Manuscripts

For peer review only

Health inequity on access to services in the ethnic minority regions of Northeastern Myanmar: A cross-sectional study

Kun Tang[1*#], **Yingxi Zhao**[2, 3#], **Bolun Li**[2], **Siqiao Zhang**[4], **Sung Hoon Lee**[5]

1 Department of Global Health, Peking University School of Public Health, Beijing, China
2 Institute for Medical Humanities, Peking University Health Science Center, Beijing, China
3 Department of Global Health, University of Washington, Seattle, WA, USA
4 Peking University School of Public Health, Beijing, China
5 Peking University School of International Studies, Beijing, China
* Correspondence: tangkun@hsc.pku.edu.cn; Tel.: +86-10-82805951;
# These authors contribute equally to the study

Kun Tang: Department of Global Health, Peking University School of Public Health, Beijing, China tangkun@hsc.pku.edu.cn
Yingxi Zhao: Department of Global Health, University of Washington, Seattle, WA, USA zhaoyingxi96@gmail.com
Bolun Li: Institute for Medical Humanities, Peking University Health Science Center, Beijing, China 450925110@qq.com
Siqiao Zhang: Peking University School of Public Health, Beijing, China 896481989@qq.com
Sung Hoon Lee: Peking University School of International Studies, Beijing, China 1400092405@pku.edu.cn

For peer review only

Abstract

Objectives: To evaluate health inequity on access to services in the ethnic regions of Northeastern Myanmar from three points of analysis: geographic barrier, gender-based disparity and financial burden of health services.

Setting: A multistage-stratified random cluster survey was conducted in Shan State Special Region 2 and Eastern Shan State Special Region 4 of Northeastern Myanmar in 2016, including a total number of 774 households.

Participants: A total number of 4,235 participants were recruited during the survey.

Primary and secondary outcome measures: geographic distance, gender, household income and inpatient/outpatient service utilization.

Results: The study results showed that residents living within 5 km of any form of healthcare facilities paid more outpatient visits (90.06 visits per thousand population) in the past two weeks, compared to those living 5-20 km and over 20 km (54.84 and 54.02 per thousand population, respectively) from healthcare facilities. A similar trend with little significant differences was found for inpatient service use. Regarding household income, adults with an annual household income of above 720 USD were more likely to seek outpatient service (OR=1.43, 95%CI 0.98-2.10) compared to those with an annual income of <360 USD. After adjusting for other covariates, female adults were less likely to seek inpatient treatment (OR=0.55, 95%CI 0.35-0.84) and outpatient services (OR=0.86, 95%CI 0.64-1.15) than male adults.

Conclusions: Geographic barrier, gender-based disparity and financial burdens were identified as key causes that significantly restrict ethnic people's access to healthcare facilities. The study concludes that tackling health inequity in Northeastern Myanmar ethnic regions requires an improved primary healthcare system, proper financial protection mechanisms and a special focus on women.

Keywords: Myanmar, conflict, health equity, ethnic minority

Strengths and limitations of this study

- The study represents the largest and one of the few population-representative studies of health inequity targeted in Myanmar-China border area, where health data is scarce,
- In terms of limitations, the cross-sectional nature of the study does not allow for causal inference to provide more robust evidence, and potential interviewer bias and respondent's recall bias may exist.

For peer review only

1. Introduction

Since Myanmar's signing of the Panglong Agreement in 1947 and its independence in 1948, the ethnic minority regions of Northeastern Myanmar have been torn apart by conflicts between the central government and ethnic armed organizations (EAOs) that seek full local autonomy. Continuous armed conflicts in Kachin, northern Shan and other states have led to the destruction of more than 200 villages and the internal displacement of some 120,000 civilians in the past years[1]. Despite the 21st Century Panglong Conference led by Aung San Suu Kyi in September 2016, which aimed to stop regional conflicts and achieve peace that has eluded the country for the past three-quarters of a century, violence in the ethnic regions has continued regardless. Recent air strikes and armed disputes across Shan State's Muse and Kutkai townships in November 2016 again resulted in dozens of casualties, with 2,600 people displaced and over 3,000 people forced to flee across the border into China [2]. The ongoing conflicts have led to a notoriously weak health status in the region: a 2014 malaria survey along the Myanmar-China border area suggested an incidence rate of 8.70 per thousand [3], almost triple the level of Myanmar's national average [4], which is all the more striking given Myanmar's already problematic health situation compared to other Southeast Asian countries in the Greater Mekong Sub-region.

Health equity has always been a key focus of public health at all levels, while issues around health equity in the conflict-ridden states of Myanmar have been especially difficult to tackle due to constraints in data. Besides nationwide health inequity, armed conflicts may also be a primary factor behind poor health status of the country, as evidenced by limited coverage of preventive intervention and high disease mortality in areas dominated by violence [5]. The infant and under-five mortality rate in Eastern Myanmar conflict regions were 94.20 and 141.90 per thousand live births in 2013, respectively, around double to triple the national average statistics of Myanmar (41.90 and 53.50 per thousand live birth in 2013, respectively) [6], and similar to the levels in Somalia [7]. Various socio-economic determinants potentially contribute to health inequity in Myanmar's conflict areas, such as geographic barriers in access to healthcare facilities, gender inequality, displacement status, financial barrier, as well as limited health management and service capacity [8].

Despite deteriorating health status in the Myanmar-China border area, the issue of health inequity in Northeastern Myanmar has largely been unnoticed in the literature. The existing literature mostly focused on the Thai-Myanmar border area, with different social and political context, ethnicity composition, economic condition and health status. Hence, this study aims to analyze health inequity in the ethnic minority regions of Northeastern Myanmar, the conflict regions of Shan State in particular, with data derived from a population-representative cross-sectional survey conducted in 2016. After evaluating the level of health service utilization in the study regions and analyzing its association with various socio-demographic indicators, this study then examines the region's high health inequity mainly from three aspects: geographic barrier, gender-based disparity and financial burden of health services.

For peer review only

2. Methods

Study Design

A survey using multistage-stratified random cluster sampling scheme was conducted in Shan State Special Region 2 (Wa region) and Eastern Shan State Special Region 4 (SR4) of Northeastern Myanmar in March, 2016. To ensure population representativeness, randomization procedures were applied through multistage sampling: at the first stage of sampling, 75 village-level units were randomly chosen with probability proportional to scale (PPS) from a sampling frame containing all village-level units under seven townships in Wa region and SR4. At the second stage, all households in a village were listed, from which ten households were randomly chosen within each village using a geographically randomized approach – after one household was picked, every other household was chosen in a geographically clockwise sequence. A total number of 774 households and 4,235 participants were eventually recruited in the survey. The household data included detailed information about the respondent family and every individual within the family. The survey questionnaire responses contained detailed information on basic demographics, household, health status, health care, employment, household economy (income, consumption and wealth), health service utilization and health knowledge. All data were collected by face-to-face computer-aided personal interviews (CAPI) and were entered into Epidata for analysis. Ethical approval was obtained from Zhejiang University. Written informed consent was obtained from all participants.

Exposure and Outcome Variables

In this study, the main outcome variables included outpatient and inpatient information. Outpatient service utilization data included the frequency of outpatient visit during the past year (none, 1, or ≥2), place of outpatient visit (i.e. private clinics, rural health centers, township hospitals, healthcare facilities in China, or others), and average cost for outpatient visits; inpatient service utilization data included the frequency of inpatient visit during the past two weeks (none, 1, or ≥2), reasons for inpatient visit (disease, poisoning and injury, delivery, or others), place of inpatient visit (private clinics, rural health centers, township hospitals, healthcare facilities in China, or others), and average cost for inpatient visits.

Exposure variables included age, gender, education level (i.e. illiterate, 0-5 years of education, ≥6 years, and under 14 years old - individuals aged below 14 were categorized as "under 14 years old" due to ongoing education), ethnicity (i.e. Dai, Anni, Wa, Lahu, Kokang, Jingpo, Burman, Blang, etc.), occupation (i.e. government/military, engineering, business and agriculture, others, and under 14 years old - individuals aged below 14 were categorized as "under 14 years old" due to ongoing education), physical distance from home to health care facilities (i.e. <5km, 5 to 20 km and >20 km), annual household income (i.e. 0 to 360 US Dollars, 360 to 720 US Dollars, and > 720 US Dollars) and general food security (i.e. enough food supply or lacking in food supply).

Statistical analysis

For peer review only - http://bmjopen.bmj.com/site/about/guidelines.xhtml

Selected demographic and socioeconomic variables were considered separately for males and females. The distribution of socio-demographic, unadjusted means with standard deviations (SD) for continuous variables and unadjusted proportions for categorical variables were represented. Health service utilization data including the frequency, reasons and places for inpatient and outpatient visit were separately considered by region and gender. The average costs for inpatient/outpatient visits were distinguished by different types of healthcare facilities.

To analyze the association between physical distance to the nearest healthcare facility and the prevalence of inpatient and outpatient visits, linear regression was performed and adjusted means of frequency to inpatient and outpatient service per thousand population by physical distance was calculated. All linear models were commonly adjusted for covariates, including age, gender, household income and ethnicity.

The association between each exposure variable and inpatient/outpatient utilizations was analyzed using logistics regression. Odds ratios (OR) of using inpatient/outpatient services were reported within each gender, income level, education level, and physical distance to nearest facility. As the population under the age of 14 did not report education level, results were separately considered for population below and above 14 years of age. All statistical analyses were performed using SAS version 9.3 (SAS Institute Inc., Cary, North Carolina, USA), and test results were reported significant at 0.05 level.

3. Results

Baseline participants' characteristics stratified by regions are presented in Table 1. Of the 4,235 participants included in the analyses, 3,246 (76.60%) were from the Wa region, and 1,638 (50.46%) in Wa region and 500 (50.56%) in SR4 were male, respectively. 41.74% of Wa region's population was under the age of 14, and 32.56% of the Wa population was between 15 and 34 years of age. In comparison, 31.75% and 34.88% of SR4 population was under 14 years and between 15 and 34 years, respectively. 44.31% and 45.91% of Wa and SR4 residents were illiterate, respectively. 29.70% of the Wa population and 73.00% of the SR4 population reported an annual income of above 720 USD, while a significant portion of the population in Wa region (44.45%) reported an annual household income of less than 360 USD. In Wa region, most of the population (82.01%) was of Wa ethnicity, while SR4 was constituted by a more complex variety of ethnicities. Half of the households in SR4 reported a physical distance to the nearest healthcare facilities within 5 km, while about 34.54% of the surveyed households in Wa region reported a physical distance to the nearest healthcare facilities over 20 km.

For peer review only

	Wa Region (N= 3,246)	Eastern Shan State SR4 (N= 989)
**Age, years**		
0-14	1355(41.74)	314(31.75)
15-34	1057(32.56)	345(34.88)
⩾35	834(25.70)	330(33.37)
**Sex**		
Male	1638(50.46)	500(50.56)
**Education**		
Under 14 years old	1355(41.74)	314(31.75)
Illiterate	1428(44.31)	454(45.91)
0-5 years	313(9.64)	147(14.86)
≥6 years	140(4.31)	74(7.48)
**Household Income**		
<360 USD	1443(44.45)	82(8.29)
360-720 USD	839(25.85)	185(18.71)
>720 USD	964(29.70)	722(73.00)
**Ethnicity**		
Wa	2662(82.01)	—
Dai	141(4.34)	352(35.59)
Aini	—	158(15.98)
Others	443(13.65)	479(48.43)
**Occupation**		
Under 14 years old	1355(41.74)	314(31.75)
Government/military	105(3.23)	37(3.74)
Engineering, business and agriculture	1670(51.45)	598(60.47)
Others	115(3.57)	40(4.04)
**Types of drinking water**		
Well water	17(0.52)	140(14.16)
Hose water	806(24.83)	685(69.26)
Others	2423(74.65)	164(16.58)
**Physical Distance to Nearest Healthcare Facility**		
<5 km	1080(33.27)	487(49.24)
5-20 km	1045(32.19)	355(35.90)
>20 km	1121(34.54)	147(14.86)
**Food Security**		
Lacking in food supply	1187(36.57)	171(17.29)

Table 1. Basic Characteristics of 4,235 Participants by Region

Source: author analysis

Values are N (%) unless otherwise specified.

Table 2 shows the characteristics of healthcare service utilization in the study regions. Only 2.16% of Wa residents and 4.65% of SR4 residents reported having received inpatient treatment in the past year. Similarly, only 6.56% of Wa residents and 6.88% of SR4 residents reported having paid outpatient visit in the past two weeks. Having disease conditions was the major reason for both inpatient and outpatient service utilizations in Wa region and SR4.

For peer review only

While more residents in Wa region preferred private clinics for outpatient services (61.03%) and township hospitals for inpatient services (45.71%), more SR4 residents chose healthcare facilities in China for inpatient service (39.13%). Of those who used health services, males were more likely to choose private clinics or health services in China. As shown in Figure 1, the average cost for outpatient and inpatient visit in China's health facilities was 449.09 USD and 1414.58 USD, respectively, which was two to three times higher than that in township hospitals (205.28 and 411.37 USD). The average cost for primary health care provided by private clinics and rural health centers was much lower, but utilization of these services remained relatively low.

	Wa Region (N=3,246)	SR4 (N=989)	*p*	Male (N=2,138)	Female (N=2,097)	*p*
**Inpatient in the past year**			<0.001			0.0180
Yes	70 (2.16)	46 (4.65)		46 (2.15)	70 (3.23)	
No	3,176 (97.84)	943 (95.35)		1092 (97.85)	2027 (96.77)	
**Reason for inpatient among all inpatient population**			<0.001			0.0402
Disease	64 (91.43)	39 (84.78)		45 (97.83)	58 (82.86)	
Poisoning and injury	2 (2.86)	1 (2.17)		1 (2.17)	2 (2.86)	
Delivery	4 (5.71)	6 (13.05)		-	10 (14.28)	
**Place for inpatient among all inpatient population**			0.0003			0.8946
Rural health centers	1 (1.43)	3 (6.52)		1 (2.17)	3 (4.29)	
Township hospitals	32 (45.71)	8 (17.40)		14 (30.43)	26 (37.14)	
Private clinics	19 (27.14)	6 (13.04)		11 (23.91)	14 (20.00)	
Healthcare facilities in China	13 (18.57)	18 (39.13)		13 (28.26)	18 (25.71)	
Others	5 (7.15)	11 (23.91)		7 (15.23)	9 (12.86)	
**Outpatient in the past two weeks**			0.7286			0.2234
Yes	213 (6.56)	68 (6.88)		132 (6.17)	149 (7.11)	
No	3,033 (93.44)	921 (93.12)		2006 (93.83)	1948 (92.89)	
**Place for outpatient among all outpatient population**			0.0003			0.2113
Rural health centers	15 (7.04)	6 (8.82)		10 (7.57)	11 (7.38)	
Township hospitals	47 (22.07)	23 (33.82)		30 (22.73)	40 (26.85)	
Private clinics	130 (61.03)	23 (33.82)		77 (58.33)	76 (51.01)	
Healthcare facilities in China	9 (4.23)	14 (20.60)		11 (8.33)	12 (8.05)	
Others	12 (5.63)	2 (2.94)		4 (3.04)	10 (6.71)	

Table 2. Characteristics of health service utilization by region and sex

Source: author analysis

Values are N (%) unless otherwise specified. P-value refers to comparison between Wa region and SR4, or between males and females

Figure 2 (a) and (b) describe the adjusted mean frequency of inpatient/outpatient visits per thousand population by physical distance. In general, residents living within 5 km paid more outpatient visits to healthcare facilities in the two weeks before the survey, compared to

For peer review only

residents living 5-20 km and over 20 km (90.06, 54.84 and 54.02 per thousand population per two weeks, respectively). In comparison, residents living within 5 km and between 5-20 km used slightly more inpatient services than did residents living over 20 km in the past year. Figure 2 (c) and (d) illustrate the gender difference in outpatient visits. For males, the average frequency of outpatient service utilization decreased from 93.15 visits per thousand population for those living within 5 km to the nearest hospital/clinic to 50.01 for those living over 20 km in males; and for females, the average frequency decreased from 83.63 for those living within 5 km to 56.96 in those living over 20 km. Shown in Figure 2 (e) and (f), SR4 population reported a larger number of outpatient visits within each distance level than those in Wa region; the mean frequency of outpatient visit for those living within 5 km was 89.85 per thousand population in the past two weeks in Wa region, while the corresponding value for SR4 was 173.09. For those living further than 20 km, the mean frequency was 59.77 and 91.57 per thousand population for Wa region and SR4, respectively.

	**Outpatient**		**Inpatient**	
	**Age**<14 OR (95% CI)	**Age≥14** OR (95% CI)	**Age**<14 OR (95% CI)	**Age≥14** OR (95% CI)
**Sex**				
Male	1.00	1.00	1.00	1.00
Female	1.06(0.66-1.72)	0.86 (0.64-1.15)	1.43 (0.55-3.73)	0.55 (0.35-0.84)
**Household Income**				
<360 USD	1.00	1.00	1.00	1.00
360-720 USD	3.43(1.73-6.83)	1.04 (0.69-1.55)	1.71 (0.51-5.76)	1.53 (0.82-2.86)
>720 USD	2.95 (1.48-5.86)	1.43 (0.98-2.10)	1.04 (0.28-3.85)	1.99 (1.09-3.64)
**Distance to nearest healthcare facilities**				
<5 km	1.00	1.00	1.00	1.00
5-20 km	0.37 (0.21-0.66)	0.67 (0.47-0.95)	0.77 (0.26-2.28)	1.07 (0.66-1.73)
>20 km	0.17 (0.07-0.38)	0.79 (0.55-1.13)	0.45 (0.12-1.74)	0.75 (0.43-1.32)
**Education**				
Illiterate	/	1.00	/	1.00
0-5 years	/	0.55 (0.35-0.86)	/	1.12 (0.66-1.90)
≥6 years	/	0.64 (0.36-1.15)	/	0.76 (0.34-1.74)
**Region**				
Wa	1.00	1.00	1.00	1.00
SR4	0.42 (0.17-1.08)	1.04 (0.64-1.68)	3.35 (0.45-25.00)	1.43 (0.75-2.71)

Table 3. Odds Ratio of the association between selected variables and health seeking behavior in Wa region and Eastern Shan State SR4*

Source: author analysis

*All models commonly adjusted for sex, household income, physical distance to nearest healthcare

For peer review only

facility, education level and ethnicity.

Table 3 shows the association between selected socio-demographic indicators and inpatient/outpatient visits. After adjusting for other covariates, females above 14 were less likely to seek inpatient treatment compared to males of the same age group (OR=0.55, 95%CI 0.35-0.84), and slightly less likely to seek outpatient treatment than males (OR=0.86, 95%CI 0.64-1.15), while no such association was found among children of both sexes aged below 14 years. Greater distance to the nearest healthcare facilities was associated with a decreased likelihood of inpatient/outpatient utilizations in all age groups. Household income was positively associated with outpatient behavior. For those aged above 14 years, those with an annual household income of above 720 USD were more likely to seek outpatient service (OR=1.43, 95%CI 0.98-2.10) compared to those with an income of less than 360 USD per year. A similar association was also observed in inpatient visits between the two income groups (OR=1.99, 95%CI 1.09-3.64). As for those aged below 14, a household income between 360-720 USD and above 720 USD both increased the odds of seeking outpatient services (OR=3.43, 95%CI 1.73-6.83, and OR=2.95, 95%CI 1.48-5.86, respectively). As for education level, longer years of education was associated with reduced odds of outpatient visits (OR=0.64 and 0.55 for those with an education of longer than 6 years and between 0-5 years, respectively).

4. Discussion

The present study is the largest and one of the few studies on health inequity in the Myanmar-China border area. Through the analysis of a population-representative survey in the Wa and SR4 regions, we found that health inequity was a major issue leading to a poor health status in the region. Such an inequity in health was attributable to poverty, lack of financially protective measures, geographic barriers and gender-based disparity.

There are several potential limitations of this study: firstly, the study's cross-sectional nature does not allow for causal inference to provide more robust evidence; secondly, the interviews were conducted by an international NGO that provided public health service to the regions, thus potential interviewer bias and respondent's recall bias need to be taken into account. Nonetheless, the present findings from the population-representative survey provides good insight into service utilization in Northeastern Myanmar border area, where health data is scarce.

a) Inequitable health due to poverty and a lack of financial protection

Our study found that socio-economic condition, represented by household income, is strongly associated with inpatient and outpatient service utilization in this region. Besides the fact that generally low income leads to low service utilization in both inpatient and outpatient services, the financial burden of health services also deters patients from accessing different tiers of healthcare facilities. People with higher household incomes tend to visit healthcare facilities

For peer review only

more frequently or use more expensive healthcare facilities in China. In contrast, most of the population with lower household income tend to use private clinics, and in most occasions, small pharmacies, where services are more likely to be provided by uncertified and unqualified health practitioners or pharmacists.

Out-of-pocket expenditure is still a major source of total health expenditure in Northeastern Myanmar border areas, and the proportion is estimated to be higher than that of central Myanmar, which was above 90% in 2014 [9]. Considering that most of the households' annual income is less than 1 dollar per day in Wa region, an outpatient visit to township hospital could cost over half of an annual household income. Besides, the local health system's bias against civilians exacerbates the problem of health inequity. Both Wa region and SR4 cover the outpatient/inpatient fee for military personnel, and reimburse 50% of the fee for soldiers' family members. However, no financial coverage is provided for civilian and children. On the other hand, in the hospitals and rural health centers opened by central Myanmar government, many health professionals charge informal payments or redirect the patients to private hospital/clinics. This poses a financial barrier for civilians in lower socioeconomic status in gaining access to government-funded healthcare services [8]. In both Myanmar government health facilities and ethnic health organizations, high fee-for-service may bring about catastrophic health care expenditures, which makes healthcare services unaffordable to patients at the lower end of the socio-economic spectrum. Tackling the financial barriers is perhaps one of the most crucial and pressing task in improving health service utilization in Northeastern Myanmar.

b) Geographic disparities

Our study found that physical distance to the nearest healthcare facilities could largely influence people's frequency of inpatient and outpatient use in the study regions. The result is consistent with most empirical studies as the patients may have difficulty accessing hospitals and clinics if the physical distance is too great [10, 11]. Lack of security due to ongoing armed conflicts in the region, degradation of transportation infrastructure and unstable external environment, including extreme weather may cause health equity to deteriorate. Nonetheless, our study suggests that geographic distance mainly influences outpatient visits, and access to primary health care in particular. There is no evidence that physical distance influences inpatient service utilization in these regions, possibly because inpatient visits are often closely correlated with severe disease conditions in which people must be transferred to health facilities for immediate medical attention.

While the lack of transportation infrastructure –people usually have to walk to the clinics especially in extreme weather conditions – largely obstructs the accessibility to healthcare facilities, uneven geographical distribution of healthcare facilities is also a major factor contributing to the inequity situation. Over 50% of the population does not have access to healthcare facilities within 5 km in both regions. There are only eight general hospitals, 60 rural health centers and less than 100 private clinics (most of them private pharmacies) in the

For peer review only

region serving the 0.6 million population. Most of these healthcare facilities are located in capital regions, leaving the suburban and rural areas in a state of vacuum in terms of access to healthcare services. In the event of an armed conflict, most of the Burmese doctors and private doctors from China would choose to suspend their services and retreat to safer regions. The unbalanced service distribution, exacerbated by armed conflicts, further aggravate the health inequity situation in the ethnic regions of Northeastern Myanmar.

c) Gender-based inequities

In the study population of those above 14 years old, females were less likely to seek inpatient treatment compared to males. Women are usually recognized as a particularly vulnerable demographic group as they constitute the majority of internally displaced population and refugees in conflict settings [8]. Rural and ethnic minority women in Myanmar, in particular, could barely achieve equal rights with men. A series of cultural, economic, legal and political factors may perpetuate gender-based inequity to healthcare services in these regions, including but not limited to cultural norms, the low social status of women, and their financial dependence on men [12]. Despite international NGO's long-term efforts on maternal and child health, very few women enjoyed hospital-facilitated child delivery, accounting for only 14.28% of all inpatient service for females, which was much lower than Myanmar's national average of 36.2% [13]. It is important to empower women in ethnic regions not only to improve the general health status but also to overcome social and cultural barriers to gender disparity.

This is one of the first studies to explore the different aspects of health inequity, including geographic barrier, gender-based disparity, as well as financial burden of health services, in the border areas of Northeastern Myanmar. The findings suggest that all these factors strongly restrict local people's access to healthcare services. Tackling health inequity in the Myanmar border areas requires a better primary healthcare system to fill the gap of geographic disparity, a better financial protection mechanism for the current health system, as well as a special focus on women and girls. However, prolonged armed conflicts not only complicate health service delivery by stressing the region's already weak health infrastructures but also worsening the gender inequity problem.

Empirical evidence suggests that countries/regions emerging from war and conflict usually end up having weak or even non-operational health systems, which further expose them to emerging and re-emerging epidemics [14]. The sole focus on vertical programs from international development assistance could serve short-term needs but may in fact further delay the strengthening of the local health system. While the national ceasefire agreement (NCA) has encountered a huge challenge [15], the nationwide peace process and the new "21st Century Panglong Conference" suggest new opportunities for health system strengthening in the ethnic regions in Northeastern Myanmar. With additional support and financial resources from national government and international agencies, a comprehensive

For peer review only

package of primary health care system should be established to reduce health inequity for ethnic peoples.

Reference

1. Weng, Lawi (2014) 200 Kachin Civilians Flee Fighting Near Hpakant. The Irrawaddy. Available: http://www.burmanet.org/news/2014/08/14/the-irrawaddy-200-kachin-civilians-flee-fighting-near-hpakant. Accessed Dec 22, 2016.
2. Myanmar Times (2016) Air Strikes Reported as Shan State Conflict Spreads. Available: http://www.mmtimes.com/index.php/national-news/23870-air-strikes-reported-as-shan-state-conflict-spreads.html. Accessed Dec 22, 2016.
3. Wang R, Dong J, Xia Z, Cai T, Zhang Q & Zhang Y, et al. (2016) Lessons on malaria control in the ethnic minority regions in northern myanmar along the china border, 2007–2014. Infectious Diseases of Poverty, 5(1), 95.
4. Mu T, Sein A, Kyi T, Min M, Aung N, & Anstey N, et al. (2016) Malaria incidence in myanmar 2005–2014: steady but fragile progress towards elimination. Malaria Journal, 15(1), 503.
5. Victora C, Fenn B, Bryce J, & Kirkwood B. (2005) Co-coverage of preventive interventions and implications for child-survival strategies: evidence from national surveys. Lancet, 366(9495), 1460.
6. Asia foundation (2015) The Long Road to recovery Ethnic and Community-Based Health Organizations Leading to Better Health in Eastern Burma. Available: http://reliefweb.int/sites/reliefweb.int/files/resources/The%20Long%20Road%20to%20Recovery%202015_Eng.pdf. Accessed Dec 22, 2016.
7. World Bank (n.d.) UN Inter-agency Group for Child Mortality Estimation, Available: https://data.worldbank.org/country/myanmar. Accessed Sept 27, 2017
8. Bornemisza O, Ranson M, Poletti T, & Sondorp E. (2010) Promoting health equity in conflict-affected fragile states. Social Science & Medicine, 70(1), 80-88.
9. World Health Organization (2014) The Republic of the Union of Myanmar Health System Review. Available: http://www.wpro.who.int/asia_pacific_observatory/hits/series/myanmar_health_systems_review.pdf. Accessed Dec 22, 2016.
10. Awoyemi T, Obayelu O, & Opaluwa H. (2010) Effect of distance on utilization of health care services in rural kogi state, Nigeria. J Hum Ecol, 35(1): 1-9.
11. Buor D. (2003). Analysing the primacy of distance in the utilization of health services in the ahafo-ano south district, Ghana. The International Journal of Health Planning and Management, 18(4), 293–311.
12. United Nations High Commissioner for Refugees (1999) Reproductive health in refugee situations: An inter-agency field manual. Available: http://www.who.int/reproductivehealth/publications/emergencies/field_manual_rh_humanitarian_settings.pdf. Accessed Dec 22, 2016.

13. Myanmar Ministry of Health (2010) Myanmar Multiple Indicator Cluster Survey 2009 – 2010. Available: https://www.unicef.org/myanmar/MICS_Myanmar_Report_2009-10.pdf. Accessed Mar 3, 2017.
14. Bisika T (2010) Health systems strengthening in conflict situations. East Afr J Public Health, 7(3), 277-281.
15. Asian Tribune (2015) Myanmar's NCA seems half-done missing some signatories Available: http://www.asiantribune.com/node/88058. Accessed Mar 3, 2017.

Contributorship statement

TK, ZYX and LBL made the statistical analysis and drafted the manuscript; ZSQ and SHL revised the article for important intellectual content and wrote part of the article. All authors read and approved the final manuscript.

Data availability

The dataset supporting the conclusion of this article is available from a third party. Please contact the corresponding author to apply to gain access to the relevant data.

Data sharing statement

No additional data available

Funding

The authors declare no funding source.

Competing interests

The authors report no conflict of interest.

Figure 1. Average cost for inpatient and outpatient visit in USD.

Source: author analysis

Figure 2. Adjust means of frequency to inpatient and outpatient service by physical distance*

Source: author analysis

*Adjusted for age, gender, household income and ethnicity

For peer review only

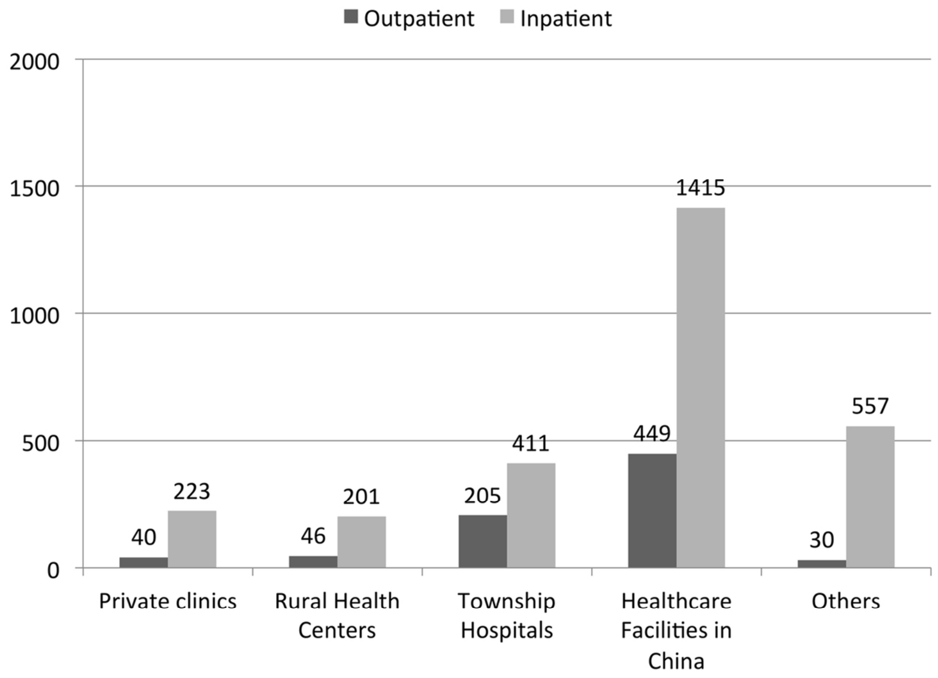

Average cost for inpatient and outpatient visit in USD

118x88mm (300 x 300 DPI)

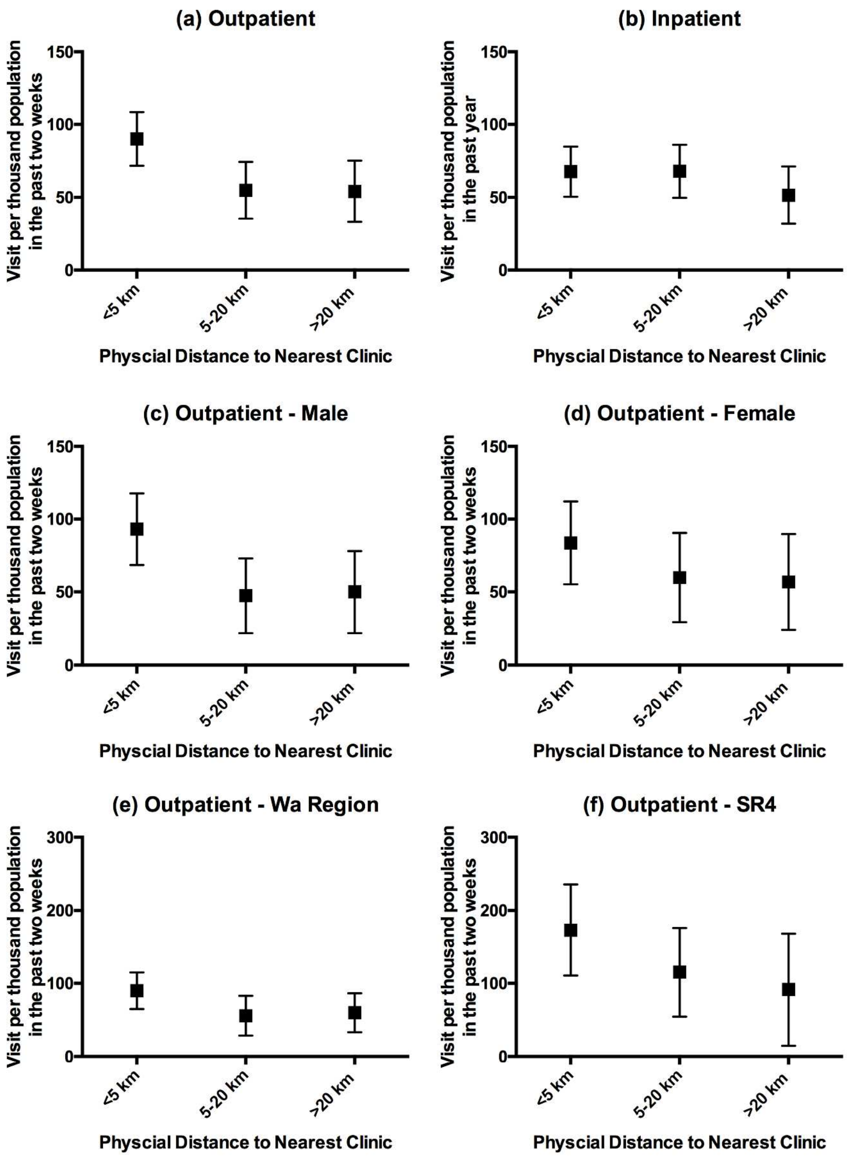

200x280mm (300 x 300 DPI)

STROBE Statement—checklist of items that should be included in reports of observational studies

	Item No.	Recommendation	Page No.	Relevant text from manuscript
**Title and abstract**	1	(*a*) Indicate the study's design with a commonly used term in the title or the abstract	1	
		(*b*) Provide in the abstract an informative and balanced summary of what was done and what was found	2	
**Introduction**				
Background/rationale	2	Explain the scientific background and rationale for the investigation being reported	3	
Objectives	3	State specific objectives, including any prespecified hypotheses	3	To analyze health inequity in the ethnic regions of Northeastern Myanmar from three aspects: geographic barrier, gender-based disparity and financial burden of health services.
**Methods**				
Study design	4	Present key elements of study design early in the paper	4	A multistage-stratified random cluster survey was conducted in Shan State Special Region 2 and Eastern Shan State Special Region 4 of Northeastern Myanmar in 2016, and a total number of 774 households were included.
Setting	5	Describe the setting, locations, and relevant dates, including periods of recruitment, exposure, follow-up, and data collection	4	A multistage-stratified random cluster survey was conducted in Shan State Special Region 2 and Eastern Shan State Special Region 4 of Northeastern Myanmar in 2016, and a total

For peer review only - http://bmjopen.bmj.com/site/about/guidelines.xhtml

				number of 774 households were included.
Participants	6	(*a*) *Cohort study*—Give the eligibility criteria, and the sources and methods of selection of participants. Describe methods of follow-up<br>*Case-control study*—Give the eligibility criteria, and the sources and methods of case ascertainment and control selection. Give the rationale for the choice of cases and controls<br>*Cross-sectional study*—Give the eligibility criteria, and the sources and methods of selection of participants	4	A total number of 4,235 participants were recruited in the survey
		(*b*) *Cohort study*—For matched studies, give matching criteria and number of exposed and unexposed<br>*Case-control study*—For matched studies, give matching criteria and the number of controls per case	N/A	
Variables	7	Clearly define all outcomes, exposures, predictors, potential confounders, and effect modifiers. Give diagnostic criteria, if applicable	4	geographic distance, gender and household income and inpatient/outpatient service utilization.
Data sources/ measurement	8*	For each variable of interest, give sources of data and details of methods of assessment (measurement). Describe comparability of assessment methods if there is more than one group	4	In this study, the main outcome variables included outpatient and inpatient information. Outpatient service utilization included the frequency of outpatient visit during the past year (none, 1, or ⩾2), place of outpatient visit (i.e. private clinics, rural health centers, township hospitals, healthcare facilities in China, or others), and average cost for outpatient visits; whereas inpatient service utilization included the frequency of inpatient visit

For peer review only - http://bmjopen.bmj.com/site/about/guidelines.xhtml

during the past two weeks (none, 1, or ⩾2), reasons for inpatient visit (disease, poisoning and injury, delivery, or others), place of inpatient visit (private clinics, rural health centers, township hospitals, healthcare facilities in China, or others), and average cost for inpatient visits.

Exposure variables included age, gender, education level (i.e. illiterate, 0-5 years of education, ⩾6 years, and under 14 years old - individuals aged below 14 were categorized as "under 14 years old" due to ongoing education), ethnicity (i.e. Dai, Anni, Wa, Lahu, Kokang, Jingpo, Burman, Blang, etc.), occupation (i.e. government/military, engineering, business and agriculture, others, and under 14 years old - individuals aged below 14 were categorized as "under 14 years old" due to ongoing education), physical distance from home to health care facilities (i.e. <5km, 5 to 20 km and >20 km), annual

For peer review only - http://bmjopen.bmj.com/site/about/guidelines.xhtml

				household income (i.e. 0 to 360 US Dollars, 360 to 720 US Dollars, and > 720 US Dollars) and family general food security (i.e. enough food supply or lacking in food supply).
Bias	9	Describe any efforts to address potential sources of bias	9	the interviews were conducted by an international NGO that provided public health service to the regions, potential interviewer bias and respondent's recall bias need to be taken into account.
Study size	10	Explain how the study size was arrived at	4	To ensure the population representativeness, randomization procedures were applied through multistage: at the first stage of sampling, 75 village-level units were randomly chosen with the probability proportional to scale (PPS) from a sampling frame containing all village-level units under seven townships in Wa region and SR4. At the second stage, all households in a village were listed, from which ten households were randomly chosen within each village using geographically randomization approach – after one household was picked, every other

For peer review only - http://bmjopen.bmj.com/site/about/guidelines.xhtml

	household was chosen in a geographically clockwise sequence.

Continued on next page

For peer review only

For peer review only - http://bmjopen.bmj.com/site/about/guidelines.xhtml

Quantitative variables	11	Explain how quantitative variables were handled in the analyses. If applicable, describe which groupings were chosen and why	4	
Statistical methods	12	(*a*) Describe all statistical methods, including those used to control for confounding	5	Selected demographic and socioeconomic characteristics were described separately for male and female. The distribution of socio-demographic, unadjusted means with standard deviations (SD) for continuous variables and unadjusted proportions for categorical variables were presented. Health service utilization including the frequency, reasons and places for inpatient and outpatient visit were separately presented by region and gender. The average cost for inpatient/outpatient visit was presented by different types of healthcare facilities.<br><br>To analyze the association between physical distance to nearest healthcare facility and inpatient and outpatient behavior, linear regression was performed and adjusted means of frequency to inpatient and outpatient service per thousand population by physical distance were presented. All linear models were commonly adjusted for covariates, including age,

For peer review only - http://bmjopen.bmj.com/site/about/guidelines.xhtml

				gender, household income and ethnicity.
		(*b*) Describe any methods used to examine subgroups and interactions	5	The association between each exposure variable and inpatient and outpatient utilizations was analyzed using logistics regression. Odds ratios (OR) of using inpatient/outpatient services were reported within each gender, income level, education level, and physical distance to nearest facility and study region. As the population under the age of 14 did not report education level, results were separately reported for population below and above 14 years of age.
		(*c*) Explain how missing data were addressed	N/A	None /
		(*d*) *Cohort study*—If applicable, explain how loss to follow-up was addressed<br>*Case-control study*—If applicable, explain how matching of cases and controls was addressed<br>*Cross-sectional study*—If applicable, describe analytical methods taking account of sampling strategy	N/A	
		(*e*) Describe any sensitivity analyses	N/A	None.
**Results**				
Participants	13*	(a) Report numbers of individuals at each stage of study—eg numbers potentially eligible, examined for eligibility, confirmed eligible, included in the study, completing follow-up, and analysed	5	
		(b) Give reasons for non-participation at each stage	N/A	None.
		(c) Consider use of a flow diagram	N/A	None.
Descriptive data	14*	(a) Give characteristics of study participants (eg demographic, clinical, social) and information on exposures and potential confounders	5 & Table 1	Baseline participants' characteristics stratified by regions are presented in Table 1. Overall, of the 4,235 participants included in

For peer review only - http://bmjopen.bmj.com/site/about/guidelines.xhtml

For peer review only

the analyses, 3,246 (76.60%) were from Wa region, and respectively 1,638 (50.46%) in Wa region and 500 (50.56%) in SR4 were male. At the time of the survey, 41.74% of the Wa region's population was under the age of 14, and 32.56% of the Wa population was between 15 and 34 years of age. In comparison, 31.75% and 34.88% of SR4 population was under 14 years and between 15 and 34 years, respectively. 44.31% and 45.91% of Wa and SR4 resident were illiterate. 29.70% of the Wa population and 73.00% of the SR4 population reported an annual income of above 720 USD, while majority of the population in Wa region (44.45%) reported an annual household income of less than 360 USD. In Wa region, most of the population (82.01%) was Wa ethnicity, while SR4 constituted a more complex variety of ethnicities. Half of the households in SR4 reported a physical distance to the nearest healthcare facilities within 5 km, while about 34.54% of the surveyed household in Wa region reported a physical distance to the nearest healthcare facilities over 20

For peer review only - http://bmjopen.bmj.com/site/about/guidelines.xhtml

For peer review only

				km.
		(b) Indicate number of participants with missing data for each variable of interest	N/A	None.
		(c) *Cohort study*—Summarise follow-up time (eg, average and total amount)		
Outcome data	15*	*Cohort study*—Report numbers of outcome events or summary measures over time		
		Case-control study—Report numbers in each exposure category, or summary measures of exposure		
		Cross-sectional study—Report numbers of outcome events or summary measures	6-7	Only 2.16% of Wa residents and 4.65% of SR4 residents reported having received inpatient treatment in the past year. Similarly, only 6.56% of Wa residents and 6.88% of SR4 residents reported having paid outpatient visit in the past two weeks. Having disease conditions was the major reason for both inpatient and outpatient service utilizations in Wa region and SR4. While more residents in Wa region preferred private clinic for outpatient services (61.03%) and township hospitals for inpatient services (45.71%), more SR4 residents chose healthcare facilities in China for inpatient service (39.13%). Of those who used health services, males were more likely to choose private clinics or health services in China. As is shown in Figure 1, average cost for outpatient and inpatient visit in China's health facilities was 449.09 USD and 1414.58 USD, respectively, which

For peer review only - http://bmjopen.bmj.com/site/about/guidelines.xhtml

				was two to three times higher than that in township hospitals (205.28 and 411.37 USD). The average costs for primary health care provided by private clinics and rural health centers were much lower, but the utility of these services were also relatively low.
Main results	16	(*a*) Give unadjusted estimates and, if applicable, confounder-adjusted estimates and their precision (eg, 95% confidence interval). Make clear which confounders were adjusted for and why they were included	6-7	Only 2.16% of Wa residents and 4.65% of SR4 residents reported having received inpatient treatment in the past year. Similarly, only 6.56% of Wa residents and 6.88% of SR4 residents reported having paid outpatient visit in the past two weeks. Having disease conditions was the major reason for both inpatient and outpatient service utilizations in Wa region and SR4. While more residents in Wa region preferred private clinic for outpatient services (61.03%) and township hospitals for inpatient services (45.71%), more SR4 residents chose healthcare facilities in China for inpatient service (39.13%). Of those who used health services, males were more likely to choose private clinics or health services in China. As is shown

For peer review only - http://bmjopen.bmj.com/site/about/guidelines.xhtml

			in Figure 1, average cost for outpatient and inpatient visit in China's health facilities was 449.09 USD and 1414.58 USD, respectively, which was two to three times higher than that in township hospitals (205.28 and 411.37 USD). The average costs for primary health care provided by private clinics and rural health centers were much lower, but the utility of these services were also relatively low.
	(*b*) Report category boundaries when continuous variables were categorized	4	annual household income (i.e. 0 to 360 US Dollars, 360 to 720 US Dollars, and > 720 US Dollars)
	(*c*) If relevant, consider translating estimates of relative risk into absolute risk for a meaningful time period	N/A	None.

Continued on next page

For peer review only - http://bmjopen.bmj.com/site/about/guidelines.xhtml

Other analyses	17	Report other analyses done—eg analyses of subgroups and interactions, and sensitivity analyses	6-7	Figure 2 (a) and (b) described the adjusted mean frequency of inpatient/outpatient visits per thousand population by physical distance. In general, residents living within 5 km paid more outpatient visits to healthcare facilities in the past two weeks, compared with residents living between 5-20 km and over 20 km (90.06, 54.84 and 54.02 per thousand population per two weeks). In comparison, residents living within 5 km and between 5-20 km used slightly more inpatient services than did residents living above 20 km in the past year. Figure 2 (c) and (d) illustrate the gender difference in outpatient visit. The average frequency of outpatient service utility decreased from 93.15 visits per thousand population in those living within 5 km to the nearest hospital/clinic, to 50.01 in those living over 20 km in males; and in females, the average frequency decreased from 83.63 in those living within 5 km to 56.96 in those living over 20 km. Shown in Figure 2 (e) and (f), SR4 population reported a larger number of outpatient visits within each

For peer review only - http://bmjopen.bmj.com/site/about/guidelines.xhtml

For peer review only

				distance level than those in Wa region, the mean frequency of outpatient visit for those living within 5 km was 89.85 per thousand population in the past two weeks in Wa region and 173.09 in SR4. For those living above 20 km, the mean frequency were 59.77 and 91.57 per thousand population for Wa region and SR4, respectively.
**Discussion**				
Key results	18	Summarise key results with reference to study objectives	9	In the analysis of a population-representative survey in two regions, we found that health inequity was a major issue leading to a poor health status in the study areas. Such an inequity in health was attributable to poverty, a lack of financial protection, geographic barriers and gender-based disparity.
Limitations	19	Discuss limitations of the study, taking into account sources of potential bias or imprecision. Discuss both direction and magnitude of any potential bias	9	There are several potential limitations of the present study: firstly, this study's cross-sectional nature does not allow for causal inference to provide more robust evidence; secondly, the interviews were conducted by an international NGO that provided public health service to the regions, potential interviewer bias and respondent's recall bias need to be taken into

For peer review only - http://bmjopen.bmj.com/site/about/guidelines.xhtml

For peer review only

				account. Nonetheless, the present findings from the population-representative survey provides a good insight of service utilization in Northeastern Myanmar border area, where health data is severely scarce.
Interpretation	20	Give a cautious overall interpretation of results considering objectives, limitations, multiplicity of analyses, results from similar studies, and other relevant evidence	11	This is one of the first studies to explore the different aspects of health inequity, including geographic barrier, gender-based disparity, as well as financial burden of health services, in border areas of northeastern Myanmar. The findings suggest that all these factors strongly restrict local people's access to healthcare services. Tackling health inequity in the Myanmar border areas requires a better primary healthcare system to fill the gap of geographic disparity, a better financial protection mechanism to build in the current health systems, as well as a special focus on women and girls. However, prolonged armed conflicts not only complicate health service delivery through the already weak health infrastructures, but also deteriorate the gender inequity.

For peer review only - http://bmjopen.bmj.com/site/about/guidelines.xhtml

Generalisability	21	Discuss the generalisability (external validity) of the study results	11	Tackling health inequity in the Myanmar border areas requires a better primary healthcare system to fill the gap of geographic disparity, a better financial protection mechanism to build in the current health systems, as well as a special focus on women and girls. However, prolonged armed conflicts not only complicate health service delivery through the already weak health infrastructures, but also deteriorate the gender inequity.
**Other information**				
Funding	22	Give the source of funding and the role of the funders for the present study and, if applicable, for the original study on which the present article is based	N/A	N/A

*Give information separately for cases and controls in case-control studies and, if applicable, for exposed and unexposed groups in cohort and cross-sectional studies.

Note: An Explanation and Elaboration article discusses each checklist item and gives methodological background and published examples of transparent reporting. The STROBE checklist is best used in conjunction with this article (freely available on the Web sites of PLoS Medicine at http://www.plosmedicine.org/, Annals of Internal Medicine at http://www.annals.org/, and Epidemiology at http://www.epidem.com/). Information on the STROBE Initiative is available at www.strobe-statement.org.

For peer review only - http://bmjopen.bmj.com/site/about/guidelines.xhtml
